# Conserved properties of genetic architecture of renal and fat transcriptomes in rat models of insulin resistance

**DOI:** 10.1242/dmm.038539

**Published:** 2019-07-15

**Authors:** Georg W. Otto, Pamela J. Kaisaki, Francois Brial, Aurélie Le Lay, Jean-Baptiste Cazier, Richard Mott, Dominique Gauguier

**Affiliations:** 1 Genetics and Genomic Medicine, University College London Institute of Child Health, 30 Guilford Street, London WC1N 1EH, United Kingdom; 2 The Wellcome Trust Centre for Human Genetics, University of Oxford, Roosevelt Drive, Headington, Oxford OX3 7BN, United Kingdom; 3 University Paris Descartes, INSERM UMR 1124, 45 rue des Saint-Pères, 75006 Paris, France; 4 Centre for Computational Biology, Medical School, University of Birmingham, Birmingham B15 2TT, United Kingdom; 5 University College London Genetics Institute, Gower Street, London WC1E 6BT, United Kingdom; 6 McGill University and Genome Quebec Innovation Centre, 740 Doctor Penfield Avenue, Montreal, QC H3A 0G1, Canada

**Keywords:** eQTL, Diabetes mellitus, Goto-Kakizaki rat, SNP, Spontaneously hypertensive rat, Systems genetics, Transcriptome

## Abstract

To define renal molecular mechanisms that are affected by permanent hyperglycaemia and might promote phenotypes relevant to diabetic nephropathy, we carried out linkage analysis of genome-wide gene transcription in the kidneys of F2 offspring from the Goto-Kakizaki (GK) rat model of type 2 diabetes and normoglycaemic Brown Norway (BN) rats. We mapped 2526 statistically significant expression quantitative trait loci (eQTLs) in the cross. More than 40% of eQTLs mapped in the close vicinity of the linked transcripts, underlying possible *cis*-regulatory mechanisms of gene expression. We identified eQTL hotspots on chromosomes 5 and 9 regulating the expression of 80-165 genes, sex or cross direction effects, and enriched metabolic and immunological processes by segregating GK alleles. Comparative analysis with adipose tissue eQTLs in the same cross showed that 496 eQTLs, in addition to the top enriched biological pathways, are conserved in the two tissues. Extensive similarities in eQTLs mapped in the GK rat and in the spontaneously hypertensive rat (SHR) suggest a common aetiology of disease phenotypes common to the two strains, including insulin resistance, which is a prominent pathophysiological feature in both GK rats and SHRs. Our data shed light on shared and tissue-specific molecular mechanisms that might underlie aetiological aspects of insulin resistance in the context of spontaneously occurring hyperglycaemia and hypertension.

## Abstract (precis)

**Summary:** Kidney and fat expression QTL mapping in rat models of spontaneously occurring insulin resistance associated with either diabetes or hypertension reveals conserved gene expression regulation, suggesting shared aetiology of disease phenotypes.

## INTRODUCTION

Transcriptome-based molecular phenotyping provides detailed information on the expression of individual genes and biological pathways. Variation in transcript abundance can be mapped to the genome at expression quantitative trait loci (eQTLs). The genetic control of gene transcription in mammals has been reported in various organs from preclinical models of human chronic diseases using experimental crosses (Davis et al., 2012; Kaisaki et al., 2016; van Nas et al., 2010), recombinant congenic strains (Hubner et al., 2005; Petretto et al., 2006), congenic strains (Dumas et al., 2016; Kaisaki et al., 2016), recombinant congenic strains (Lee et al., 2006) and mice of the collaborative cross (Aylor et al., 2011). Mapping of eQTLs in humans has progressed from whole blood and cell systems (Dixon et al., 2007; Fairfax et al., 2012; Grundberg et al., 2012) to multiple postmortem organs in control individuals (Battle et al., 2017), which were used to identify genes and biological pathways causing chronic diseases through computational integration with genome-wide association study (GWAS) data (Gamazon et al., 2018).

However, this strategy does not account for expected organ-specific variation in gene expression in disease conditions, which requires access to biopsies from the affected tissues that are often impossible to collect in large groups of phenotypically homogeneous patients and healthy control subjects. In addition, the genetic basis of several diseases remains poorly characterized through GWAS. Diabetic nephropathy is a typical example of a complex genetic disease condition where causative genes have not been mapped robustly in humans through GWAS (Ahlqvist et al., 2015) and where the identification of contributing molecular mechanisms is therefore essential. These limitations underline the importance of preclinical models of human chronic diseases to define tissue-specific genetic control of gene transcription accurately in standardized experimental conditions.

The Goto-Kakizaki (GK) rat is a model of type 2 diabetes mellitus, which spontaneously exhibits biochemical and histological evidence of nephropathy (Janssen et al., 2004). It was produced over many generations of breeding outbred Wistar rats using glucose intolerance as the sole criterion for selecting breeders (Goto et al., 1976) in order to enrich the genome of offspring in naturally occurring polymorphisms contributing to impaired glucose homeostasis progressively, while integrating alleles promoting increased susceptibility, and possibly resistance, to diabetes endophenotypes and associated complications (Bihoreau et al., 2017). We and others have derived intercrosses between GK and normoglycaemic Brown Norway (BN) rats to map comprehensively the genes responsible for the control of glucose tolerance, insulin secretion and adiposity (Gauguier et al., 1996), islet morphology (Finlay et al., 2010), lipid metabolism (Argoud et al., 2006), proteinuria (Nobrega et al., 2009), plasma metabolomic variables (Dumas et al., 2007) and adipose tissue gene transcription (Kaisaki et al., 2016).

Here, we characterized the genetic architecture of renal gene expression in chronic diabetes through the mapping of kidney eQTLs in a GK×BN F2 cross. We identified similarities in the genetic architecture of gene transcription regulation in kidney and adipose tissue and conserved eQTL genes in the GK rat and the spontaneously hypertensive rat (SHR), suggesting common molecular mechanisms and shared genetic aetiology of pathophysiological phenotypes. These data provide detailed information on genomic regulation in the diabetic kidney, which can point to individual genes and biological pathways involved in the aetiopathogenesis of human diabetic nephropathy and insulin resistance.

## RESULTS

### Genetic mapping of genome-wide gene transcription control in the diabetic kidney in (GK×BN) F2 rats

We have previously shown that adult rats (7 months old) of our GK colony exhibit varying degrees of thickening of the glomerular basement membrane and mild mesangial extracellular matrix expansion when compared with normoglycaemic BN control animals (Wallis et al., 2008). To characterize the architecture of renal gene transcription that might account for these renal structural changes and for proteinuria that segregates in a GK×BN F2 cross (Nobrega et al., 2009), we applied an eQTL strategy to map genetic loci linked to quantitative variations in the abundance of renal transcripts in the GK×BN F2 offspring previously used for analysis of diabetes QTLs (Gauguier et al., 1996). Genotypes at >255 framework markers typed in the cross were used to impute allele probabilities and construct a map of 898 marker positions (2.5 cM spacing between markers). These were used to test for linkage to detectable Illumina array signals in each of the 123 F2 rats, as previously described (Kaisaki et al., 2016). Genome sequencing of the GK/Ox strain (Atanur et al., 2013) allowed us to exclude data from Illumina oligonucleotides for 757 genes containing genetic polymorphisms between GK and BN (Kaisaki et al., 2016), which might alter binding between probes and the oligonucleotides (Alberts et al., 2007), resulting in spurious eQTLs or questionable lack of genetic linkage to the corresponding transcripts. As previously observed in adipose tissue eQTLs mapped in the same cross (Kaisaki et al., 2016), >40% of probes with DNA polymorphisms gave significant eQTL signals [logarithm of odds (LOD)>10], which deviated from the distribution eQTL significance when all oligonucleotides were considered (Fig. 2). After filtering out probes with low intensity signal, a total of 15,759 transcripts were considered for linkage analyses.

We identified a total of 2526 eQTLs at false discovery rate (FDR)<0.05 (Table 1), including 2252 (89%) linked to transcripts localized to a single genomic position in the rat genome assembly (RGSC3.4, Ensembl release 69) (Table S1). Analysis of eQTL statistical significance showed that 509 eQTLs (20.1%) could be detected with a high level of confidence (LOD>10) (Fig. 3A; Table S1). The chromosome distribution of eQTLs showed that on average 11% of genes on each chromosome were regulated by eQTLs in the cross (Table 1). An excess of eQTLs was detected on chromosomes 5 (27% of genes were associated with an eQTL) and 9 (23% of genes were associated with an eQTL) (Table 1).

**Table 1.**
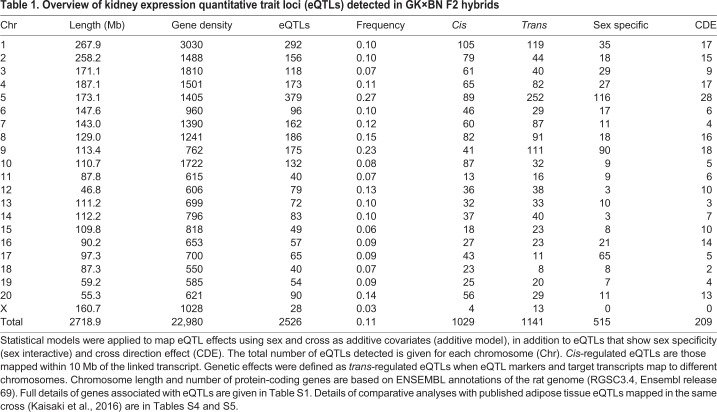
**Overview of kidney expression quantitative trait loci (eQTLs) detected in GK×BN F2 hybrids**

### Genetic mapping of local and distant renal gene transcription regulation in (GK×BN) F2 rats

To assess gene transcription regulation through *cis-* and *trans*-mediated genetic effects, we compared the genomic positions of transcripts and linked marker loci showing the strongest evidence of significant linkage (Fig. 3B). A total of 1141 eQTLs (46%) accounted for linkage between markers and transcripts mapped to different chromosomes, which unambiguously underlie *trans*-mediated mechanisms of gene transcription regulation (Table 1; Table S1). The remaining 1356 eQTLs mapped to chromosomes where the linked transcripts were encoded, including 1283 eQTLs linked to genes assigned to a unique position in the rat genome assembly. A high proportion of these eQTLs were within 10 Mb (*n*=1029, 80.3%) or 5 Mb (*n*=788, 61.4%) of the linked transcripts, which could underlie *cis*-regulatory mechanisms of gene transcription (Fig. 3B; Table S1). The mean LOD score for statistically significant eQTLs was 8.32±8.65 (s.d.), and there was an inverse relationship between eQTL significance and frequency (Fig. 3C). eQTLs localized in the close vicinity of the linked transcripts, which are most likely to be regulated in *cis*, showed the highest proportion of statistically significant linkage (Fig. 3D). Polygenic control of the expression of 133 genes was suggested when two or more eQTLs were linked to the same gene (Table S1).

Kidney eQTLs were generally distributed evenly across the genome (Fig. 3E). However, we noted an excess of *trans*-mediated eQTLs on chromosomes 5 (*n*=252) and 9 (*n*=111), which accounts for the above-mentioned high number of eQTLs on chromosomes 5 (*n*=379) and 9 (*n*=175) (Table 1). This can be explained by eQTL clustering in regions of chromosomes 5 (50-55 cM; 120.3-132.3 Mb) and 9 (79.5-84.5 cM; 99.2-104.4 Mb), which control the expression of 165 genes (chromosome 5 eQTLs) and 80 genes (chromosome 9 e QTLs) generally regulated in *trans* (Fig. 3E; Table S1). GK rat genotypes at the eQTL hotspot on chromosome 5 were predominantly associated with downregulated expression of an important proportion of eQTL genes (129/165; 78%), suggesting that this phenomenon might have genuine biological relevance and reflect the existence of a master regulator of gene transcription at this locus.

### Genetic analysis of the renal gene transcriptome identifies sex and cross direction effects

**Fig. 1.**
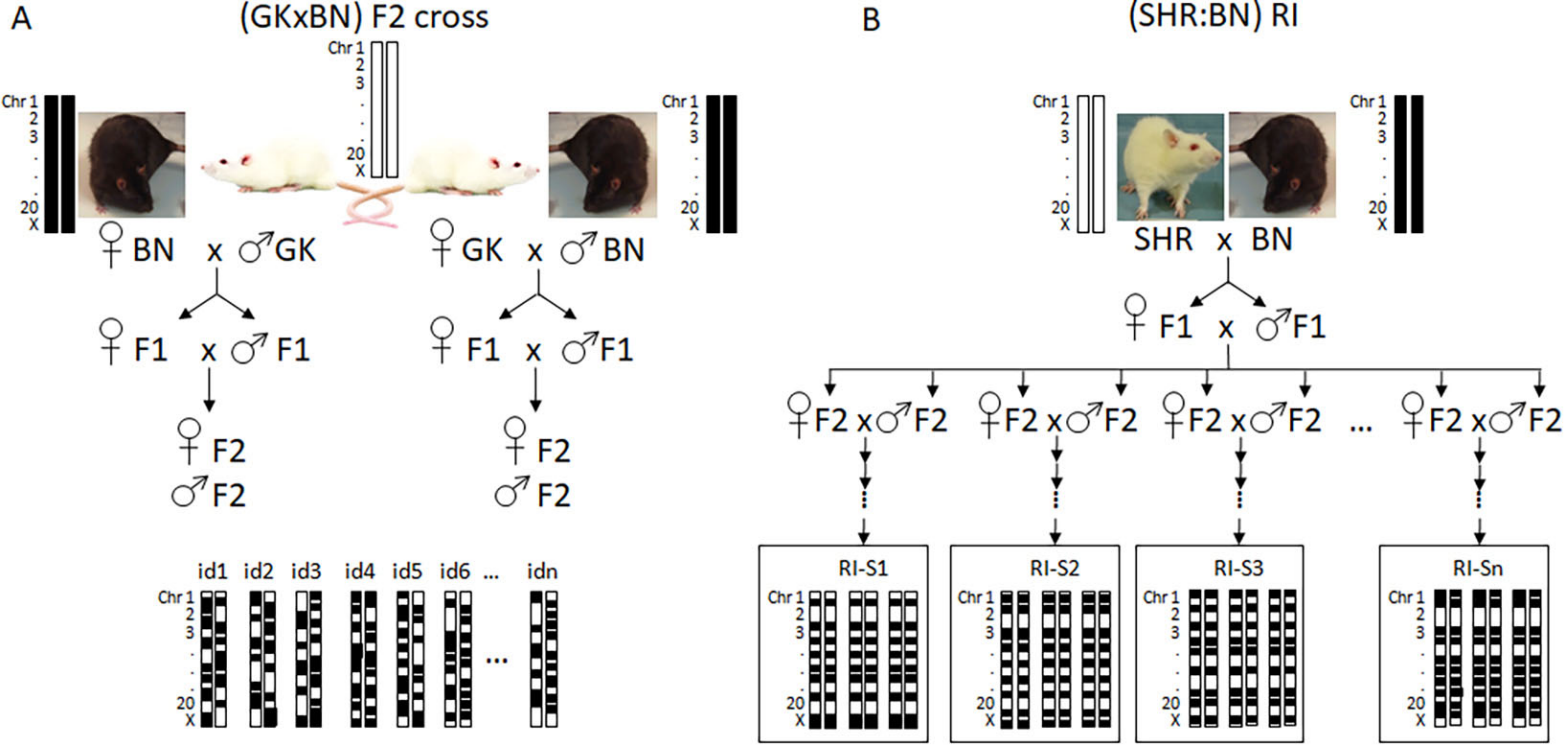
**Breeding design applied to map expression quantitative trait loci (eQTL) in the Goto-Kakizaki (GK) rat strain.** (A,B) Reciprocal genetic crosses between male and female GK and Brown Norway (BN) rats used to produce individual (GK×BN) F2 rats are illustrated (A) and compared with the breeding scheme designed to derive recombinant inbred strains (RI-S) from spontaneously hypertensive rats (SHRs) and BN rats (B).

The design of the experimental cross combining male and female F2 hybrids originating from GK females or GK males (Fig. 1) allowed us to detect sex and cross direction-specific patterns of gene expression for 516 and 209 kidney eQTLs, respectively (Table 1; Table S2). eQTLs showing cross direction effects (CDEs) were evenly distributed across the genome (Fig. 3F,G), with an average frequency of 0.9% of genes impacted by CDEs, and few were highly significant (maximal interactive LOD=20.4) (Table 1; Table S2). Although parent-of-origin effects, which might account for parental imprinting, cannot be assessed in our experimental setting of an F2 cross, several eQTLs showing evidence of CDEs in the GK×BN F2 cross were linked to imprinted genes, including *Igf2* (LOD=4.58, *P*=0.001), *Ndn* (LOD=4.81, *P*<0.001), *Zfat* (LOD=5.52, *P*<0.001), *Tp53* (LOD=4.85, *P*<0.001) and *Cdk4* (LOD=2.93, *P*=0.049). Interestingly, expression of *Igf2*, *Ndn* and *Tp53* was regulated by CDE eQTLs in the same region of chromosome 2 (98.9-103.9 cM), suggesting the existence of a regulator of gene imprinting in this region.

**Fig. 2.**
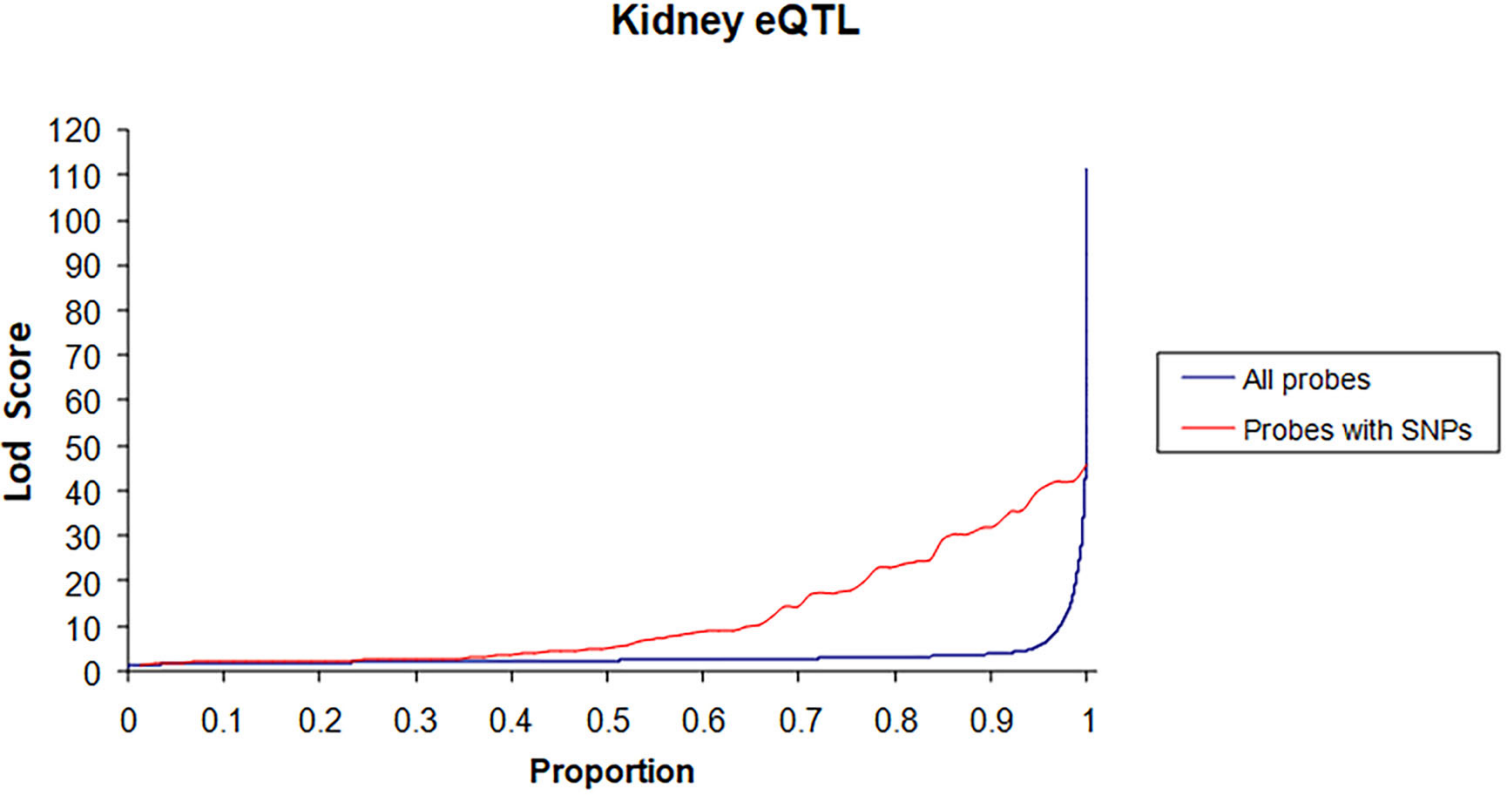
**Distribution LOD scores for renal expression quantitative trait loci (eQTL) mapped in the GK×BN F2 cross.** LOD scores are plotted against the proportion of eQTLs detected with all Illumina oligonucleotides (blue line) and those containing DNA variation between GK and BN strains (red line).

In contrast to CDE eQTLs, instances of strongly significant linkages were found for sex-specific eQTL effects (maximal interactive LOD=104.7) (Table S2). Sex-specific and CDE eQTLs were mutually exclusive. Only one locus on chromosome 4 exhibited both sex-specific effects (LOD=3.04) and CDE (LOD=2.92) *trans*-mediated linkage with the gene *Trak2*. Few eQTLs detected in the full F2 population combining both males and females showed evidence of significant CDEs (*Akr1c2*, *Dbndd1*, *F2* and *Ptpro*). By contrast, a relatively large number of sex-specific eQTLs, generally regulated in *cis*, were also significant in the full F2 population (*Akr1b7*, *Ank2*, *Armc3*, *Ifitm6*, *LOC494499*, *LOC500300*, *LOC684289*, *Neurl2*, *Nfkbia*, *Pou4f1*, *RGD1309350*, *RGD1559948*, *RGD1562107*, *RGD1563820*, *RGD1564419*, *RGD1564696*, *RGD1565387*, *RGD1566401*, *Scgb1c1*, *Slc22a13*, *Sparcl1*, *Spink8* and *Tgfb2*).

**Fig. 3.**
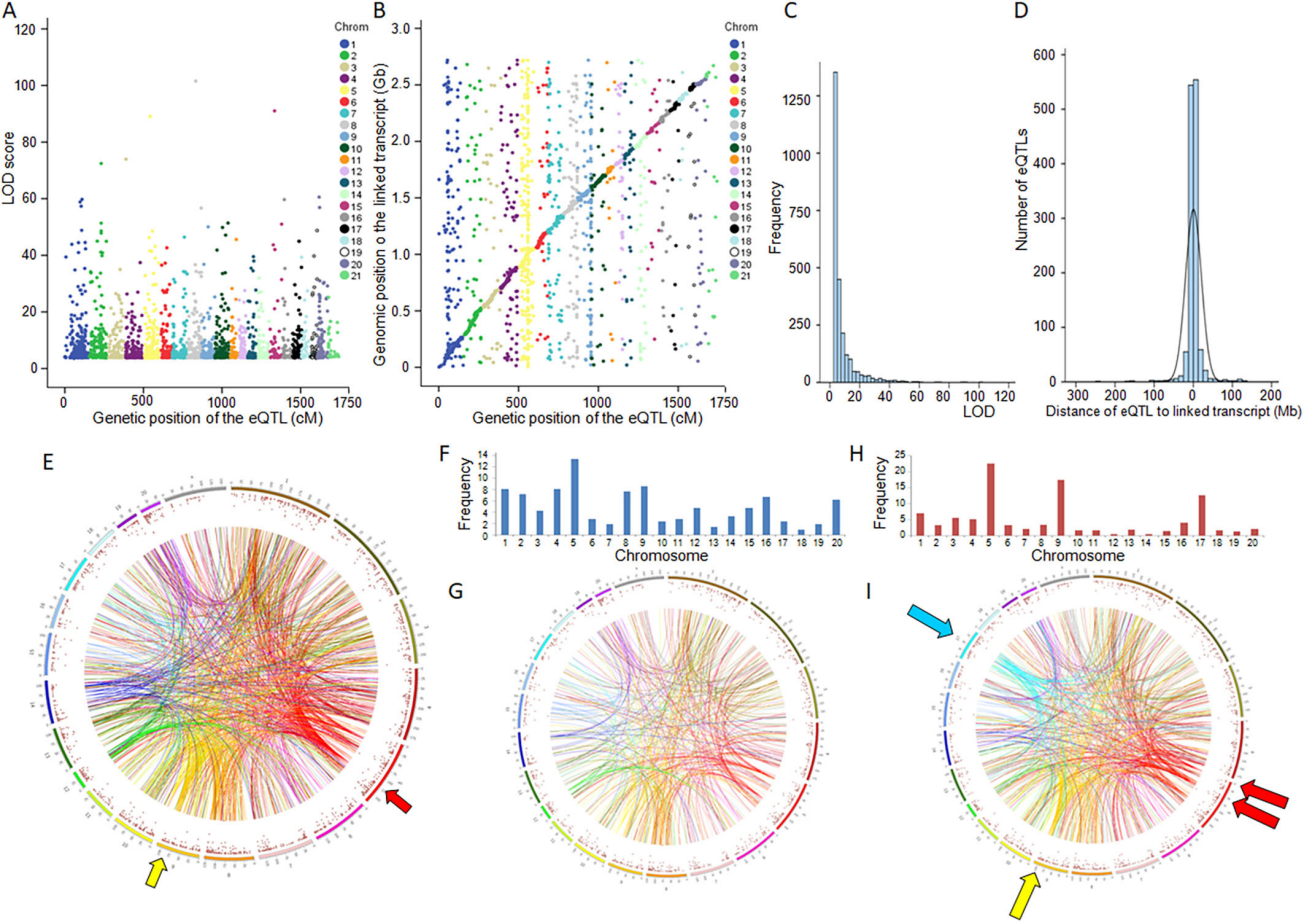
**Kidney expression quantitative trait loci (eQTL) architecture in GK×BN F2 hybrids.** (A) Genetic positions of statistically significant eQTLs (FDR *P*-value <0.05) are plotted against the LOD scores. (B) Local and distant eQTLs are illustrated by plotting genetic positions of statistically signiﬁcant eQTLs and the genomic position of the linked transcripts. (C) Distribution of LOD scores for significant eQTLs is shown. (D) Genome mapping of pairs of eQTL and linked transcripts localized in the same chromosomes was used to determine relationships between the statistical significance of genetic linkage and genomic distances between transcripts and genetic markers. (E) Mapping data from pairs of transcripts and eQTLs localized to different chromosomes illustrate distant (*trans*) effects of genetic loci on gene transcription and eQTL hotspots (arrows). (F-I) Chromosomal distribution and genome-wide *trans*-mediated regulation of cross direction effect (CDE) (F,G) and sex-specific (H,I) eQTLs are shown. Chromosomes are colour coded on the circle to illustrate the effects of eQTLs mapped to the same chromosomes on the expression of distant genes. Arrows indicate genomic regions of eQTL enrichment. Details of eQTLs are given in Tables S1 and S2.

The vast majority of eQTLs showing CDEs were unambiguously mediated in *trans* (Fig. 3G), whereas the most significant sex-specific eQTLs (LOD>10) were predominantly linked to genes mapped to the same chromosome (25/30; 83%) and often localized in the close vicinity (<10 Mb) of linked transcripts (19/30; 63%), suggesting *cis*-mediated regulation of gene expression (Table S2). Sex-specific eQTLs were found on all rat chromosomes (Fig. 3H), with an average of 2.2% showing this effect across the genome (Table 1). However, when the gene density of an individual chromosome was considered, we noted a much stronger proportion of genes showing a sex-specific eQTL pattern on chromosomes 5 (*n*=116; 8.3%), 9 (*n*=90; 11.8%) and 17 (*n*=65; 9.3%) (Table 1; Table S2). Overall, 52.6% of sex-specific eQTLs were localized on chromosomes 5 (22.5%), 9 (17.5%) and 17 (12.6%) (Table 1; Table S2; Fig. 3H), and they were organized in clusters in the centromeric and interstitial regions of chromosome 5 (0-2.5 cM, 0-5.8 Mb, *n*=27 and 25-32.5 cM, 53.1-68.2 Mb, *n*=53), at the telomeric end of chromosome 9 (69.9-84.5 cM, 90.5-104.4 Mb, *n*=71) and in the interstitial regions of chromosome 17 (36.1-48.6 cM, 53.0-80.2 Mb, *n*=45) (Fig. 3I; Table S2). GK alleles at the sex-specific eQTLs in the centromeric region of chromosome 5 (0-2.5 cM) were predominantly associated with upregulated gene expression (21/27), whereas they were associated with downregulated gene expression on chromosome 9 (47/71).

### eQTL-based pathway analysis underscores the involvement of immunological processes and drug metabolism in the diabetic kidney

To identify biological consequences of diabetes and segregating GK/BN polymorphisms across the rat genome on renal transcriptional changes, we carried out pathway enrichment analysis of genes under eQTL control in F2 hybrids. A total of 40 biological pathways were significantly altered, including primarily mechanisms related to drug and xenobiotic metabolism by cytochrome P450 and also various immunological functions covering autoimmune diseases (thyroid and graft versus host diseases, type 1 diabetes and rheumatoid arthritis) and cellular immune and inflammatory processes (e.g. antigen processing and presentation, phagosome) (Table 2). Metabolic pathways were also significantly affected, including bile acids, pentose and glucuronate, amino acids (alanine, aspartate, glutamate, valine, leucine, isoleucine and selenocompounds), arachidonic acid, butanoate, ketone bodies and vitamins (ascorbate, vitamin B6, retinol and folate). Altered processes related to cell adhesion, O-glycan biosynthesis and nitrogen metabolism might contribute to impaired regulation of renal structure and function.

**Table 2.**
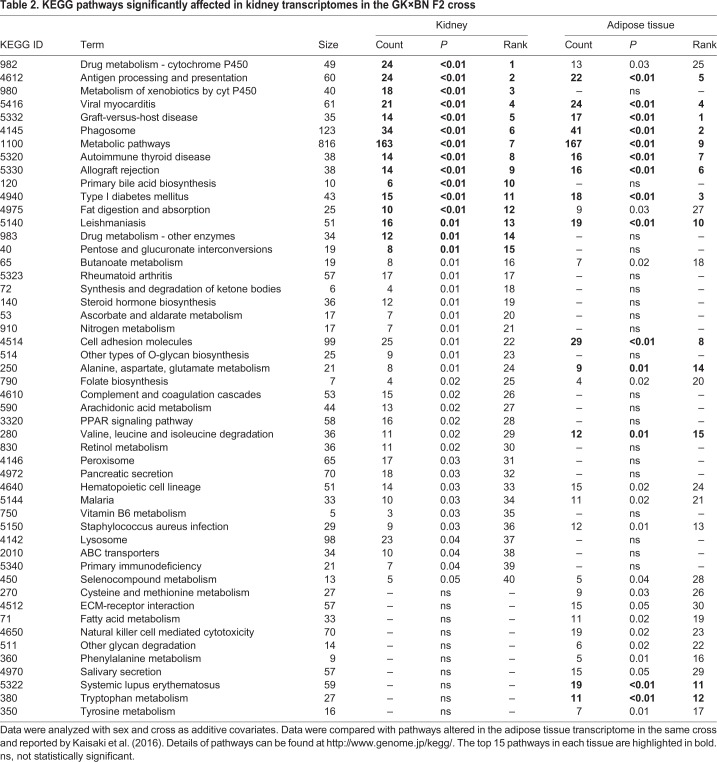
**KEGG pathways significantly affected in kidney transcriptomes in the GK×BN F2 cross**

eQTL genes contributing to the top ranking pathway (drug metabolism, KEGG 982) included aldehyde oxidases (*Aox1* and *Aox3l1*), cytochrome P450 (*Cyp2c11*, *Cyp2d1*, *Cyp2d4*, *Cyp2d5* and *Cyp2e1*), flavin-containing monooxygenases (*Fmo1*, *Fmo2*, *Fmo4* and *Fmo5*), glutathione *S*-transferases (*Gsta4*, *Gsta5*, *Gstm2*, *Gstm4*, *Gstp1*, *Gstt1* and *Gstt2*) and UDP glucuronosyltransferases (*Ugt1a1*, *Ugt1a7c*, *Ugt2b10*, *Ugt2b15*, *Ugt2b17* and *Ugt2b37*) (Table S1). The eQTL genes for these glutathione *S*-transferases and several UDP glucuronosyltransferases also accounted for the enrichment of the metabolism of xenobiotics by the cytochrome P450 pathway (KEGG 980), and eQTL genes for these cytochromes P450 and these UDP glucuronosyltransferases contributed to the retinol metabolism pathway (KEGG 830).

eQTLs linked to RT1 class I and II genes (*RT1-A2*, *RT1-CE5*, *RT1-CE10*, *RT1-CE15*, *RT1-CE16*, *RT1-Da*, *RT1-DMa*, *RT1-DMb*, *RT1-Ha*, *RT1-M3-1*, *RT1-M10-1*, *RT1-N2*, *RT1-N3* and *RT1-CE5*) dominated the enrichment of the antigen processing and presentation pathway (KEGG 4612). eQTLs for all these RT1 genes, along with *RT1-T24-4*, lysosomal H^+^-transporting ATPases (*Atp6ap1*, *Atp6v0a4*, *Atp6v0e1*, *Atp6v1d* and *Atp6v1g3*) and tubulins (*Tuba4a*, *Tubb2b*, *Tubb3* and *Tubb5*) accounted for the enrichment of the phagosome pathway (KEGG 4145).

The results from our genome-wide functional analysis of the genetic control of renal gene transcription provide a comprehensive landscape of altered biological functions in the diabetic kidney and underline the high level of complexity of coordinated regulation of molecular processes contributing to renal pathological mechanisms in diabetes in the GK rat.

### Kidney and adipose tissue transcriptomes exhibit tissue-specific eQTL features

**Fig. 4.**
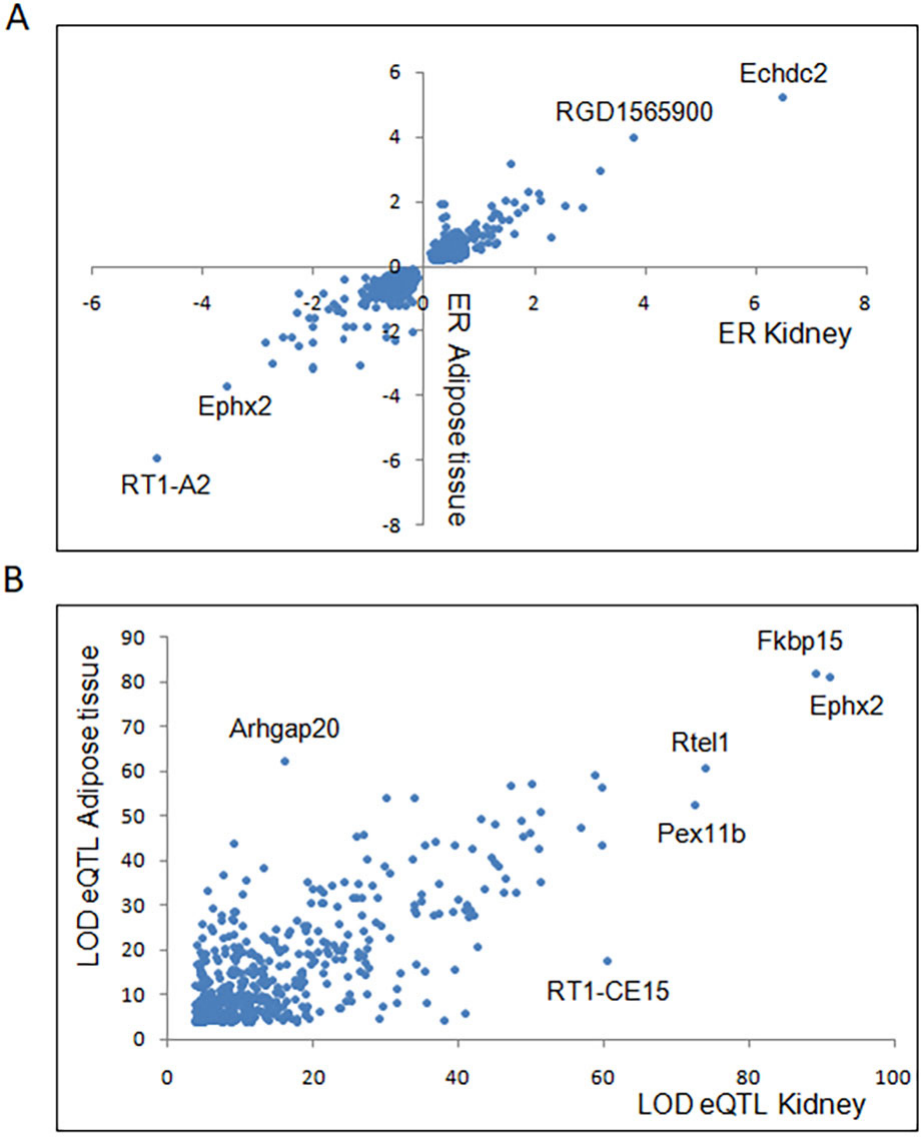
**Comparative analyses of kidney and adipose tissue expression quantitative trait loci (eQTLs) mapped in the GK×BN F2 cross.** (A,B) The effect of GK alleles at the eQTLs [expression ratio (ER)] on gene expression (A) and eQTL statistical significance (LOD score) (B) in kidney and adipose tissue were plotted to illustrate the strong conservation of eQTLs detected in both tissues. Full details of statistically significant eQTLs, illustrating shared and tissue-specific genetic regulation of gene expression, are given in Table S4.

To investigate biological functions consistently affected in different tissues in diabetes in the GK rat, we compared architectural and functional features of kidney eQTLs with previously reported adipose tissue eQTLs mapped in animals of the same GK×BN F2 cross using strictly identical experimental and analytical procedures (Kaisaki et al., 2016). We identified approximately the same number of eQTLs in kidney (2526) and adipose tissue (2735) in the cross (Table S3). Hotspots of eQTLs mostly mediated in *trans* were identified in both tissues, but they were detected on different chromosomes in adipose tissue (chromosomes 1, 7 and 17) and kidney (chromosome 9), and in different regions of chromosome 5 in adipose tissue (57.5–62.5 cM) and kidney (0-2.5 and 25-32.5 cM). At the gene level, nearly 20% (496/2526, *P*=6×10^−4^, one-sided Fisher's exact test) of predominantly locally regulated (*n*=414) kidney eQTLs were also significant in adipose tissue in the cross (i.e. eQTL mapped within 15 cM in the two tissues and consistent allelic effect on gene expression changes), which reveal conserved molecular mechanisms in the two tissues. There were good correlations between kidney and adipose tissue for both the magnitude of the allelic effects (Fig. 4A) and LOD scores (Fig. 4B) at the common eQTLs. By contrast, only a few genuine *trans*-mediated eQTLs were conserved in the two tissues (Table S4).

Sex effects and CDEs were also observed in adipose tissue eQTLs (Table S3). However, we did not identify a large excess of sex-specific adipose tissue eQTLs on any chromosome, in contrast to our observations on chromosomes 5 and 9 in kidney. In the vast majority of cases, sex-specific and CDE eQTLs identified in the adipose tissue trancriptome were mutually exclusive (Table S5). Only 10 genes showed both sex effects and CDEs (*Gper*, *Olr241*, *LOC501102*, *LOC501113*, *Ppic*, *Ppp4c*, *RGD1564266*, *Rtp3*, *Setdb2* and *Wdr24*), but only two (*Gper* and *LOC501113*) were controlled by the same locus. Conserved sex- and cross direction-specific genetic effects in fat and kidney were observed for only 11 and 10 genes, respectively, but the location of the eQTLs was consistent for only two genes (*Nfkbia* and *Rai14*) for sex effects and one gene (*Tarsl2*) for cross direction effects (Tables S2 and S5).

### eQTLs drive tissue-specific functional enrichment in kidney and adipose tissue

To identify organ-specific and shared genetic regulation of biological pathways in diabetes transcriptomes, we compared results from gene set enrichment analysis of kidney and adipose tissue eQTLs mapped in the GK×BN F2 cross. Eleven of the top 15 biological pathways regulated by eQTLs in kidney were also significantly altered in adipose tissue (Table 2). We found evidence of conserved biological effects of eQTLs in the two tissues for immunological functions, including autoimmune diseases (e.g. thyroid and graft versus host diseases, type 1 diabetes) and autoimmune or inflammatory mechanisms (e.g. antigen processing and presentation, phagosome, allograft rejection), and for the metabolism of drugs by cytochrome P450 and metabolism of butanoate, selenocompounds and several amino acids (alanine, aspartate, glutamate, valine, leucine and isoleucine). By contrast, enrichment of eQTLs for genes involved in the metabolism of ketone bodies, steroid hormones, nitrogen, arachidonic acid, retinol and vitamins B6 and C was significant specifically in the kidney, whereas enrichment of eQTLs for genes involved in the metabolism of fatty acids and amino acids (cysteine, methionine phenylalanine, tryptophan and tyrosine) was specific to the adipose tissue.

**Fig. 5.**
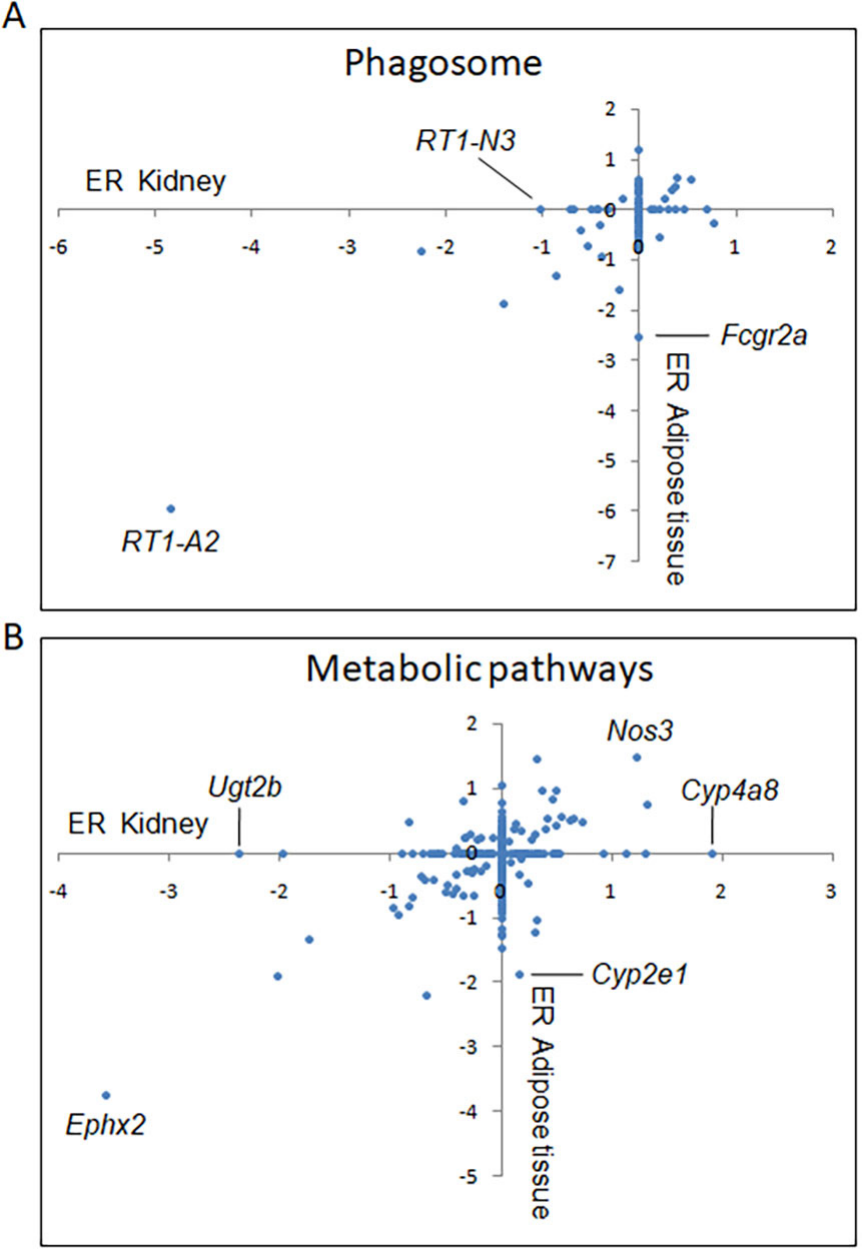
**Tissue-specific contribution of expression quantitative trait loci (eQTLs) genes to pathways enriched in both kidney and adipose tissue.** (A,B) The expression ratio (ER) of eQTLs genes contributing to significant enrichment of phagosome (A) and metabolic pathways (B) in kidney and white adipose tissue are plotted to illustrate tissue-specific and conserved effects of segregating GK alleles in the GK×BN F2 cross on eQTL gene transcription regulation. Full details of statistically significant eQTLs, illustrating shared and tissue-specific genetic regulation of gene expression, are given in Table S4.

Common transcriptional regulation of the phagosome pathway (KEGG 4145) in both kidney and adipose tissue was mostly driven by shared strongly significant *cis*-eQTLs (LOD>7) linked to *RT1-A2*, *RT1-CE5*, *RT1-CE10*, *RT1-CE15*, *RT1-CE16*, *RT1-M3-1*, *RT1-T24-4*, *Cd36* and *Dync1li1* (Fig. 5A). Other strongly significant eQTL genes contributing to the enrichment of this pathway were detected only in kidney (*RT1-N3*, *Atp6v0a4*, *Ctss*, *Tubb2b* and *Tlr6*) or in adipose tissue (*Fcgr2a*, *RT1-CE12*, *Clec4m* and *Atp6v1c1*). This pattern of overlapping but largely incomplete conservation of eQTL regulation of biological pathways between tissues was also observed for the metabolic regulation pathway (KEGG 1100) (Fig. 5B). Consistent genetic regulation of metabolic regulation was based on only 38 consistent eQTLs in kidney and adipose tissue that co-localized in the genome and showed the same allelic effects on the direction of gene expression changes in the two tissues. The remaining eQTLs contributing to the enrichment of metabolic pathways were localized to different chromosomes, showed discordant gene expression patterns in the two tissues (*n*=22), or were detected specifically in kidney (*n*=123) (e.g. *Ugt2b*, *Cyp4a8*) or in fat (*n*=114) (e.g. *Cyp2e1*) (Table S4).

The valine, leucine and isoleucine degradation pathway (KEGG 280) provided a typical example of the complexity of tissue-specific genetic control of gene transcription (Table 3). Although the pathway showed consistent significant enrichment in both kidney and adipose tissue, with similar numbers of genes contributing in the two tissues, the eQTL genes responsible for the enrichment showed clear tissue-specific expression. Only the eQTL gene *Bckdhb* showed a conserved mapping position and consistent genetic regulation of expression in kidney and adipose tissue. All other eQTL genes were either tissue specific or mapped to different chromosomal locations in the two tissues.

**Table 3.**
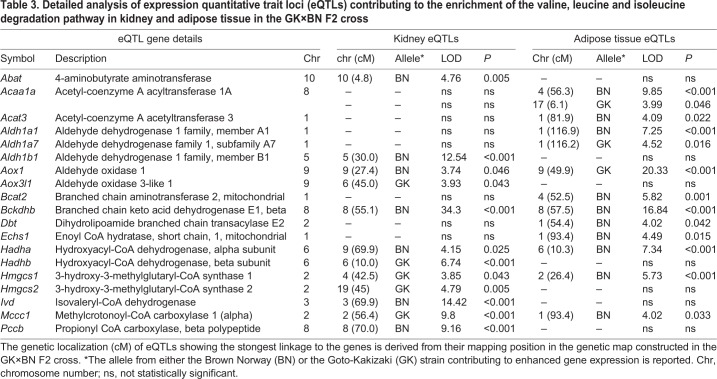
**Detailed analysis of expression quantitative trait loci (eQTLs) contributing to the enrichment of the valine, leucine and isoleucine degradation pathway in kidney and adipose tissue in the GK×BN F2 cross**

These data illustrate organ-specific genetic regulation of gene transcription patterns and the involvement of distinct molecular components regulating the same pathway in different tissues in the context of type 2 diabetes and its renal complications.

### Kidney and adipose tissue eQTLs mapped in the GK rat are conserved in the SHR

To test the pathophysiological relevance of our findings, we compared eQTLs mapped in the GK×BN F2 cross with those identified in a rat recombinant inbred (RI) panel derived from the SHR and the BN strain previously used to map eQTLs in adipose tissue and kidney (Hubner et al., 2005). Both the GK rat and the SHR are models of insulin resistance that derive from a similar outbred stock of Wistar rats selected for glucose intolerance (GK rat) (Goto et al., 1976) or high blood pressure (SHR) (Gauguier, 2005). eQTL comparisons in the two mapping systems were based on renal and adipose tissue eQTLs identified in the SHR:BN RI for 255 (kidney) and 203 (fat) known genes. Of these, 56 kidney eQTL genes (22%) and 52 adipose tissue eQTL genes (26%) were conserved in the BN×GK F2 cross (Table S6). The effect of GK and SHR alleles on gene transcription was consistent for 38 kidney eQTLs (68%) and 36 adipose tissue eQTLs (69%), suggesting shared genetic control of the linked genes in the two strains. By contrast, *trans*-regulated eQTLs identified in RI strains almost systematically mapped to a different chromosome in the F2 cross and therefore underlie distinct genetic regulation (Table S6).

To test eQTL consistency in GK rats and SHRs further, we considered genes linked to genetic markers localized in a window of 10 Mb in the two mapping systems, in order to account for differences in mapping resolution of genetic markers in an F2 cross and in RI strains. Using this criterion, we identified 24 kidney eQTLs and 17 fat eQTLs that showed evidence of co-localization in the two systems (Table 4). Of these, the majority of eQTLs were *cis*-mediated in kidney (*n*=19; 79%) and fat (*n*=13; 76%), and three were ambiguous in the F2 cross. We identified only two *trans*-mediated eQTLs (*Nrd1* and *Sectm1b*) conserved in GK rats and SHRs. Five genes (*Ascl3*, *Cd36*, *Dcps*, *Ilf3* and *Mrpl4*) were linked to eQTLs in the two tissues. In the vast majority of cases, the effects of the GK and SHR alleles on the expression of the linked genes were consistent.

**Table 4.**
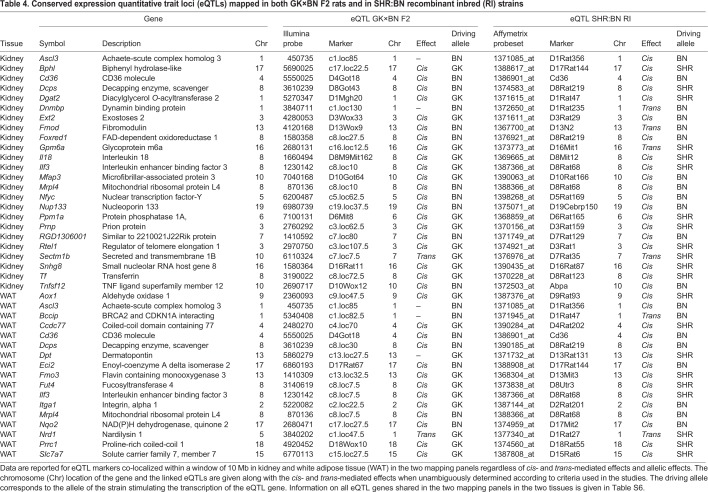
**Conserved expression quantitative trait loci (eQTLs) mapped in both GK×BN F2 rats and in SHR:BN recombinant inbred (RI) strains**

These data underline similarities in the genetic regulation of gene transcription in the GK rat and the SHR and suggest common genetic aetiology of insulin resistance in both models.

## DISCUSSION

We have characterized the genetic architecture and function of the renal transcriptome in diabetes in an F2 cross derived from diabetic GK rats and normoglycaemic BN control animals, which we compared with adipose tissue eQTL data in the cross and transcriptome data in a panel of recombinant inbred (RI) strains derived from rats of the SHR model of spontaneous hypertension associated with insulin resistance and BN control rats. We provide mapping and functional details of shared and organ-specific genetic regulation of gene expression in kidney and adipose tissue in the F2 cross. Similarities in the genetic control of gene transcription in the GK rat and the SHR suggest shared disease aetiology in these models and provide information on molecular mechanisms and genes involved in insulin resistance and renal anomalies in the context of chronic hypertension and hyperglycaemia.

Linkage mapping in the cross identified instances of polygenic control of renal transcripts, eQTL hotspots and sex-specific effects and CDEs, which were mainly distinct from those previously reported in adipose tissue in the same cross (Kaisaki et al., 2016). Several of these eQTL features, which are dominated by transcription regulation in *trans*, have been described in many species and experimental settings (Albert et al., 2018; Hasin-Brumshtein et al., 2016; Kaisaki et al., 2016; Tian et al., 2016). Parent-of-origin effects on gene expression cannot be disentangled in F2 progenies and cannot unambiguously explain CDEs on eQTLs. Evidence of increased severity of diabetes in F1 progeny of GK mothers (Gauguier et al., 1994) and the regulation of imprinted genes by CDE eQTLs in the GK×BN F2 cross suggest that parental imprinting affects the phenotypes and gene expression in the GK rat.

The low effect size of *trans*-mediated eQTLs is an important issue in assessing genuine sex-specific and CDE eQTLs. The impact of CDEs on eQTLs has not been addressed in the context of an F2 cross. By contrast, genotype by sex effects have been documented in QTL studies, including in the GK×BN F2 cross (Gauguier et al., 1996), and the general consensus is that they have a modest effect on eQTLs (Dimas et al., 2012; Kassam et al., 2016), as on many physiological QTLs (Krohn et al., 2014). The definition of *cis*-mediated gene expression regulation and eQTL hotspots, which relies on arbitrary estimates of physical distances between genetic markers and linked transcripts (Breitling et al., 2008; Petretto et al., 2006), is problematic in experimental crosses and RI strains owing to the extensive linkage disequilibrium that prevents the high resolution mapping required to separate closely linked eQTLs. Nevertheless, our present assessment of renal eQTL hotspots and *cis*-regulated eQTLs is consistent with our previous eQTL mapping data in adipose tissue in the GK×BN F2 cross, which we validated in congenic series from the same strain combination (Kaisaki et al., 2016).

Data from our previous Illumina microarray-based renal transcriptome analyses in the GK rat (Hu et al., 2009; Wilder et al., 2009) provide experimental validation for a large number of kidney eQTLs. About 44% (*n*=589) of genes differentially expressed in the kidney between GK and BN rats are controlled by eQTLs in the F2 cross. These figures are consistent with our adipose tissue transcriptome data, which showed that 43% (531/1221) of differentially expressed genes between GK and BN rats corresponded to eQTLs (Kaisaki et al., 2016). Even considering the extreme phenotypic and genomic divergence between GK and BN rats (Atanur et al., 2013; Saar et al., 2008) potentially contributing to gene expression changes, as much as 26% (*n*=347) of genes differentially expressed in kidney between GK and Wistar-Kyoto strains (Hu et al., 2009), which are derived from the same outbred Wistar stock and are therefore closely related genetically, are renal eQTLs in the GK×BN F2 cross.

Genome-wide transcriptome profiling allows unbiased and systematic analyses of biological pathways. Pathways related to immunological processes, which are believed to play a role in type 2 diabetes (e.g. phagosome, cell adhesion molecules, natural killer cell-mediated cytotoxicity), were significantly altered in both kidney and adipose tissue in the cross. We also noted associations with metabolic pathways (bile acids, ketone bodies, steroid hormones, arachidonic acid, leucine, isoleucine and valine) relevant to mechanisms involved in type 2 diabetes and potentially contributing to pathophysiology in GK rats. In particular, the branched-chain amino acids (BCAAs) leucine, isoleucine and valine are essential amino acids synthesized by gut bacteria (Amorim Franco and Blanchard, 2017) and are associated with insulin resistance (Newgard et al., 2009) and increased risk of type 2 diabetes (Lotta et al., 2016). The identification of largely distinct series of eQTL genes independently contributing to BCAA metabolism in kidney and adipose tissue in the GK×BN F2 cross demonstrates the tissue-specific genetic control of a biological function involved in diabetes. It suggests that different components of the pathway are expressed in the two tissues or that the pathway is fully expressed in the two tissues, but different components are under eQTL control.

At the gene level, our eQTL data provide information on the genetic control of transcription of individual genes that might contribute to phenotypes relevant to type 2 diabetes that we and others have mapped to the rat genome in F2 crosses and congenic strains (Bihoreau et al., 2017). We detected eQTLs for genes known to carry functional variants contributing to glucose intolerance QTLs in the GK rat, including the inositol polyphosphate phosphatase-like 1 (*Ship2* and *Inppl1*) and the insulin degradation enzyme (*Ide*) (Fakhrai-Rad et al., 2000; Marion et al., 2002). Of direct relevance to the present study, our renal eQTL data can assist in the identification of genes underlying QTLs for proteinuria detected in a GK×BN F2 cross (Nobrega et al., 2009). The 1.5 LOD confidence intervals around the peaks of maximal linkage of these QTLs (approximately 77-120 Mb on chromosome 5 and 60-112 Mb on chromosome 7; RGSC3.4, Ensembl release 69) contain a total of 69 renal eQTLs in our GK×BN F2 cross, which represent functional and positional candidates for these proteinuria QTLs in the GK rat. Among these, the 16 *cis*-regulated eQTL genes localized at the proteinuria QTLs (*Abca1*, *Ambp*, *Angptl3*, *Cdk5rap2*, *Echdc2*, *Fkbp15*, *Fktn*, *Inadl*, *Laptm4b*, *Ly6c*, *Mysm1*, *Nipsnap3b*, *RGD1311188*, *RGD1564131*, *Rraga* and *Slc22a22*) are particularly relevant because sequence variants at the loci alter their expression. Of note, the glycosyltransferase fukutin (*Fktn*, eQTL LOD=8.02, *P*<0.001) regulates the number and structure of podocytes (Kojima et al., 2011), and the α-1-microglobulin/bikunin precursor (*Ambp*, eQTL LOD=14.53, *P*<0.001) is elevated in the urine of patients with diabetic nephropathy (Zubiri et al., 2014). The identification of renal eQTLs for genes involved in polycystic kidney disease (*Pkd2*, *cis*-effect, LOD=19, *P*<0.001; *Pkdrej*, *trans*-effect, LOD=4.88, *P*=0.0042) provides molecular evidence of the extent of renal anomalies in the GK rat.

We identified similarities in eQTLs mapped in the GK rat and the SHR. Both strains exhibit insulin resistance and renal anomalies and share an important proportion of genetic polymorphisms (Atanur et al., 2013; Saar et al., 2008), which were isolated following a process of phenotype-based selection of outbred Wistar rats. We tested the hypothesis of conserved genetic regulation of transcription in GK rats and SHRs by comparing eQTLs mapped in kidney and adipose tissue in both GK×BN F2 rats and SHR:BN RI strains. Similar numbers of eQTLs were detected in the two tissues in F2 and RI rats (Grieve et al., 2008). In both tissues, we identified a high concordance of eQTLs in GK rats and SHRs despite differences in the following: (1) maintenance conditions of the animals; (2) platforms used for transcriptome analyses (Illumina beadchips in F2 rats, Affymetrix arrays in RI strains); (3) statistical methods applied to eQTL mapping; and (4) genotype frequencies (three genotypes in the F2 cross and either SHR or BN homozygous genotypes at all loci in RI). The last of these resulted in differences in genetic mapping resolution in the F2 and RI panels, making comparisons of *cis*- and *trans*-mediated effects in the two populations difficult.

In strong support to our hypothesis of common genetic factors contributing to disease phenotypes in GK rats and SHRs or SHR-related strains (SHR Stroke Prone and Spontaneously Hypertensive Heart Failure), we identified eQTLs in the GK rat for three genes that are central to SHR aetiopathogenesis, including the angiotensin I converting enzyme (*Ace*), which was the strongest candidate in the initial genetic study in the SHR (Hilbert et al., 1991), the Cd36 protein (Aitman et al., 1999) and the epoxide hydrolase 2 (*Ephx2*), which is associated with heart failure (Monti et al., 2008). We previously reported evidence of massive downregulated renal expression of *Ephx2* in the GK rat when compared with both BN rats (expression fold change: −11.7, *P*=2.7×10^−26^) and Wistar-Kyoto rats (expression fold change: −9.1, *P*=1.1×10^−24^) (Hu et al., 2009). We demonstrate here the strong significance of the genetic control of its expression in the GK×BN F2 cross (LOD=91.1), where the GK alleles at the *Ephx2* locus are associated with downregulated *Ephx2* transcription. Furthermore, we identified renal eQTLs in the GK×BN F2 cross for genes directly related to *Ephx2* function, including *Ephx1* (LOD=4.3) and *Ephx4* (LOD=5.2), where the GK alleles are associated with downregulation of transcription. According to the mitigating effects of *Ephx2* inactivation on renal injury induced by hyperglycaemia in mice (Bettaieb et al., 2017), inhibition of its expression in the GK rat might underlie compensatory mechanisms to prevent renal dysfunction.

eQTL genes in the GK rat provide functional information relevant to the pathogenesis of nephropathy in type 2 diabetes and insulin resistance in humans. Compared with other chronic diseases, large-scale genetic studies of diabetic kidney disease have provided few consistent clues and replicated genes underlying the cause of the disease, even in the largest population study (van Zuydam et al., 2018). This situation underscores the importance of functional data derived from preclinical models. Knowledge of synteny conservation between rat and human genomes (Wilder et al., 2004) can be used to exploit renal eQTL data produced in the rat to annotate the function of positional candidate genes in genomic regions linked to diabetic nephropathy in humans (Imperatore et al., 1998). Renal eQTL data in the GK rat can also provide direct functional information on risk genes for kidney disease phenotypes identified through GWAS. Associations with glomerular ﬁltration rate were identified for the human homologues of renal eQTL genes encoding the DEAD box polypeptide 1 (*Ddx1*) (*cis*-mediated, LOD=10.89, *P*<0.001), dipeptidase 1 (*Dpep1*) (*trans*-mediated, LOD=3.97, *P*=0.039), jun D proto-oncogene (*Jund*) (*cis*-mediated, LOD=7.01, *P*<0.001), LDL receptor-related protein 2 (*Lrp2*) (*cis*-mediated, LOD=5.38, *P*=0.003), myopalladin (*Mypn*) (*trans*-mediated, LOD=4.08, *P*=0.027), neuregulin 1 (*Nrg1*) (*trans*-mediated, LOD=4.76, *P*=0.009), origin recognition complex subunit 4 (*Orc4*) (LOD=4.07, *P*=0.027), tyrosine phosphatase receptor O (*Ptpro*) (*cis*-mediated, LOD=13.43, *P*<0.001) and xylulokinase, (*Xylb*) (*cis*-mediated, LOD=8.25, *P*<0.001) (Morris et al., 2019; Pattaro et al., 2016). The association with PTPRO was significant in diabetic individuals. Suggestive association was also reported between the human homologue of the *trans*-mediated renal eQTL gene *Abcc8* (LOD=4.47, *P*=0.007) and diabetic nephropathy in patients with type 2 diabetes (Jeong et al., 2019). Finally, the involvement of EPHX2 in diabetic nephropathy was suggested in a case-control study in type 2 diabetic patients (Ma et al., 2018).

Interestingly, loci associated with IgA nephropathy in humans (Kiryluk et al., 2014; Yu et al., 2011; Zhou et al., 2014) contain homologues of kidney eQTL genes in the GK rat encoding integrin alpha X (*Itgax*) (*cis*-regulated, LOD=8.86, *P*<0.001), myotubularin related protein 3 (*Mtm3*) (*trans*-regulated, LOD= 3.95, *P*=0.04) and tumor necrosis factor 13 (*Tnfsf13*) (*cis*-regulated, LOD=10.87, *P*<0.001), suggesting that the GK strain exhibits a broad spectrum of disease phenotypes and that the GK eQTL data can contribute to enhance knowledge of molecular mechanisms involved in a wide range of human disorders.

### Conclusions

GWASs of diabetic kidney disease have yet to deliver consensus causative genes with a strong effect size accounting for its genetic aetiology in humans. Our data provide original information on altered genetic control of renal genes and biological pathways in a preclinical model of type 2 diabetes, which spontaneously develops renal anomalies relevant to diabetic nephropathy. Conserved genetic control of transcription in the kidney in diabetic GK rats and in the SHR model of hypertension associated with insulin resistance, in particular for genes causing disease phenotypes in the SHR, suggests common disease aetiopathogenesis in the two strains. Our data might therefore have broad applications in the definition of molecular targets and pathways of renal structural and functional anomalies in humans beyond diabetic nephropathy.

## MATERIALS AND METHODS

### Animals

The GK×BN F2 cross (*n*=123) previously derived to map QTLs for pathophysiological phenotypes (Gauguier et al., 1996), metabolomic variables (Dumas et al., 2007) and adipose tissue eQTLs (Kaisaki et al., 2016) was used for kidney eQTL mapping. The cohort consisted of 60 males and 63 females generated from two reciprocal crosses of 55 F2 rats originating from a GK female and 68 F2 rats originating from a BN female (Fig. 1). At 6 months, animals were fasted overnight and killed by terminal anaesthesia. The right kidney was isolated, snap frozen in liquid nitrogen and stored at −80°C until RNA preparation.

### RNA preparation

Total RNA was prepared from 100 mg of frozen kidney using the RNeasy^®^ 96 Universal Tissue kit (Qiagen, Crawley, UK) as previously described (Kaisaki et al., 2016). Frozen tissue samples were homogenized in QIAzol Lysis Reagent using Qiagen's Tissue Lyser. Total RNA was purified and eluted in 90 µl of RNase-free water. RNA concentrations were determined using a NanoDrop spectrophotometer, and RNA integrity was assessed using an Agilent 2100 Bioanalyser (Agilent Technologies, Waldbronn, Germany).

### Illumina Bead Array hybridization, scanning and data processing

Kidney gene transcription profiling was performed as previously described (Kaisaki et al., 2016) using Sentrix^®^ BeadChipRatRef-12v1 Whole-Genome Gene Expression Arrays (Illumina Inc., San Diego, CA, USA), which contain 22,523 oligonucleotide probes (replicated on average 30 times), allowing quantification of transcript levels for 21,910 genes. Double-stranded cDNA and purified biotin-labelled cRNA were synthesized from 300 ng high quality total RNA using the Illumina^®^ TotalPrep RNA amplification kit (Ambion Inc., Austin, TX, USA). cRNA concentrations were determined using a NanoDrop spectrophotometer, and cRNA quality and integrity were assessed on an Agilent 2100 Bioanalyser (Agilent Technologies, Waldbronn, Germany). A total of 750 ng of each biotinylated cRNA was used for hybridization onto the arrays. BeadChip arrays were scanned on the Illumia^®^ Bead Array Reader (Illumina Inc.), and data were analysed using the Illumina^®^ Bead Studio Application software before undergoing comprehensive statistical analysis. Quality control parameters were: Gsat (green saturation) with 0≤Gsat≤1; Green 95th percentile (GP95) for consistency between arrays (∼2000 intensity units); Green 5th percentile (GP5) background level in the range of ∼100 units or below. Before array analysis, data from 757 Illumina oligonucleotides, which carry DNA variants between GK and BN rats (Kaisaki et al., 2016), were removed. We also withdrew probes that detected only background signal (i.e. Illumina detection score <0.5 in >50% of samples). Microarray data processing was carried out using the normexp background correction and quantile normalization (Shi et al., 2010).

Microarray experiments were compliant with MIAME (Minimum Information About a Microarray Experiment), and both protocol details and raw data have been deposited in ArrayExpress (http://www.ebi.ac.uk/arrayexpress/) under the accession number E-MTAB-969.

### Genetic mapping of expression QTLs in the F2 (GK×BN) cross

eQTL analysis was performed using the R-qtl software package (Broman et al., 2003) as previously described (Kaisaki et al., 2016) based on genetic maps constructed in the cross (Wilder et al., 2004). The Haley-Knott regression method was used for genome scans (Haley and Knott, 1992). We used sex and cross direction as additive covariates in our models to account for effects of sex and lineage on gene expression (Solberg et al., 2004). Permutation tests (*n*=1000) were carried out to determine the genome-wide significance threshold for each transcript. QTLs with a genome scan adjusted *P*-value <0.05 were considered significant.

The regression model with sex and cross direction as additive covariants was as described by Kaisaki et al. (2016). To detect sex×genotype interactions, QTL mapping analyses were also carried out using regression models containing sex as interactive covariates (model *H_int_*):
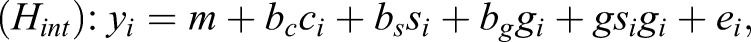
with *y_i_* denoting the phenotype (expression level of a gene) in individual *i*, *m* the baseline (mean) expression level, *b_c_c_i_* the effect of the cross, *b_s_s_i_* the effect of sex, *b_g_g_i_* the effect of the genotype, *gs_i_g_i_* the interaction of sex
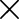
genotype and *e_i_* the residual error. To obtain evidence for interactions between QTLs and sex, *H_int_* was compared to the additive model *H_add_*. This results in *LODf*:
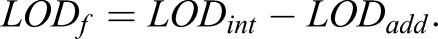


To detect cross×genotype interactions (CDEs), we included cross direction as an interaction term.

### Pathway analyses

Pathway analyses of transcriptome data were performed as previously described (Kaisaki et al., 2016) to determine functional categories enriched in the eQTL-controlled genes in the F2 cross. A hypergeometric test was used on gene ontology terms and KEGG pathways associated with gene sets against the background of genes with detectable expression (Falcon and Gentleman, 2007).

## Supplementary Material

Supplementary information
