# Supplementary material for: Conserved properties of genetic architecture of renal and fat transcriptomes in rat models of insulin resistance
Source: Dis Model Mech. 2019 Jul 15;12(7):dmm038539. doi: 10.1242/dmm.038539 (PMC6679378; doi:10.1242/dmm.038539)
Supplement: Supplementary information [file dmm-12-038539-s1.pdf]

Table S1. Details of kidney eQTLs mapped in the GKxBN F2 cross. The additive model taking sex and cross into account was used to assess eQTL statistical significance. Cis-regulated eQTLs correspond to evidence of significant linkages between markers mapped within 10Mb to the linked transcript. The position (Mb) of the genes is based on ENSEMBL annotations of the rat genome (RGSC3.4, Ensembl release 69).

Click here to Download Table S1

Table S2. Details of renal eQTLs mapped in the GKxBN F2 cross showing statistical evidence of sex or cross direction effects. The position of the markers (cM) and linked genes (bp) are based on the genetic map constructed in the cross and ENSEMBL annotations of the rat genome (RGSC3.4, Ensembl release 69). chr, chromosome.

Click here to Download Table S2

Table S3. Comparative analysis of eQTLs detected in the kidney and adipose tissue in GKxBN F2 hybrids. Statistical models were applied to map eQTL effects using sex and cross as additive covariates (additive model), as well as eQTLs that show sex-specificity (Sex interactive) and cross direction effect (CDE). The total number of eQTLs detected is given for each chromosome. Cis-regulated eQTL are those mapped within 10Mb of the linked transcript. Genetic effects were defined as trans-regulated eQTLs when eQTL markers and target transcripts map to different chromosomes. Shared eQTLs were significant in both kidney and fat and showed conserved transcriptional effects of GK alleles at the eQTL. Chromosome length and number of protein-coding genes are based on ENSEMBL annotations of the rat genome (RGSC3.4, Ensembl release 69). Full details of genes associated with kidney eQTLs are given in Supplementary Table 2. Details of the sex-specific eQTLs and CDE eQTLs in the adipose tissue are supplementary table 6. Chrom, Chromosome. * Data published in Kaisaki et al., G3 6(11):3671-3683, 2016.

Click here to Download Table S3

Table S4. Details of comsistent eQTLs mappedin kidney and adiposet tissue in the GKxBN F2 cross. The additive model taking sex and cross into account was used to assess eQTL statistical significance. Cis-regulated eQTLs correspond to evidence of significant linkages between markers mapped within 10Mb to the linked transcript. The position (Mb) of the genes is based on ENSEMBL annotations of the rat genome (RGSC3.4, Ensembl release 69).

Click here to Download Table S4

Table S5. Details of adipose tissue eQTLs mapped in the GKxBN F2 cross showing statistical evidence of sex or cross direction effect. The position (Mb) of the markers and linked genes are based on ENSEMBL annotations of the rat genome (RGSC3.4, Ensembl release 69). Cis-regulated eQTLs correspond to evidence of significant linkages between markers mapped within 10Mb to the linked transcript. eQTL genes showing evidence of both sex or cross direction effect are highlighted in green. NS, Not statistically significant.

Click here to Download Table S5

Table S6. Comparative analysis of kidney and adipose tissue eQTLs mapped in the GKxBN F2 cross and in SHR:BN recombinant inbred strains

Click here to Download Table S6
